# B cells and humoral immunity in melanoma: regulatory and autoimmune-like features and implications for immunotherapy

**DOI:** 10.1080/2162402X.2026.2638620

**Published:** 2026-03-06

**Authors:** Lucy Booth, Madeena Mahmood, Xinyi Chen, Joanna Jacków-Malinowska, Sophia Tsoka, Thomas J. Tull, Sophia N. Karagiannis

**Affiliations:** a St. John's Institute of Dermatology, School of Basic & Medical Biosciences & KHP Centre for Translational Medicine, Guy's Hospital, King's College London, London, United Kingdom; b Department of Informatics, Faculty of Natural, Mathematical and Engineering Sciences, King's College London, London, United Kingdom; c Breast Cancer Now Research Unit, School of Cancer & Pharmaceutical Sciences, King's College London, Innovation Hub, Guy's Hospital, London, United Kingdom

**Keywords:** B cell, antibody, humoral immunity, antibody, autoantibody, melanoma, immunotherapy, checkpoint inhibitors, regulatory B cell, autoimmunity

## Abstract

B cells and the humoral immune response are increasingly recognized as critical modulators of melanoma progression and immunotherapy outcomes. While checkpoint inhibitor (CPI) therapy was developed to target T cell exhaustion mechanisms, emerging evidence highlights the complexity and clinical relevance of B cell biology in this highly immunogenic cancer. Dysregulated B cell subsets, including enriched circulating naïve and immunosuppressive populations, and skewing toward immune-inert antibody isotypes such as IgG4, correlate with diminished Fc-mediated effector functions and poor survival. Regulatory B cells (Bregs) contribute to immune tolerance by inducing regulatory T cells (Tregs) and shaping the suppressive tumor microenvironment (TME) via the secretion of immunosuppressive cytokines (TGFβ and IL-10). While intratumoral B cells exhibit clonal expansion, somatic hypermutation, and polyreactivity, the expression of antibodies with high frequencies of unproductive sequences may support an active yet aberrant autoimmune-like humoral response. Conversely, mature class-switched memory B cells and tumor-resident B cell populations, including those assembled in tertiary lymphoid structures (TLSs), are associated with improved CPI responses. Dynamic changes in circulating B cell phenotypes and autoantibody profiles during CPI treatment further link humoral immunity to therapeutic efficacy and immune-related adverse events (irAEs). Collectively, these findings underscore a dual role for B cells in melanoma, supporting antitumor immunity or promoting immune escape, and highlight opportunities to target Bregs, correct isotype imbalance, and leverage B cell signatures as biomarkers. Monitoring humoral responses before and during CPI therapy may inform patient stratification, predict toxicity, and guide interventions to optimize immunotherapy outcomes.

## Introduction

Melanoma, the most aggressive type of skin cancer, presents significant therapeutic challenges. The removal of early-stage melanomas through surgical excision has been the standard treatment; however, surgery alone is often insufficient to control advanced-stage disease, and patients therefore require subsequent systemic therapies. Historically, chemotherapy and radiotherapy have had limited success in extending long-term survival in patients with metastatic disease.^1^

Despite its aggressive clinical presentation, melanoma has been considered a highly immunogenic tumor, that is able to express neoantigens that are likely to stimulate a baseline systemic and peripheral immune response, and infiltration of immune cells into tumors.^2^^,^^3^ However, melanoma evades immune surveillance, reducing immune cell activity, potency, and preventing effective disease clearance. Immunosuppressive mechanisms include the induction of regulatory, exhausted pathways on T cells, including checkpoints such as programmed death ligand 1 (anti-PD-L1) and cytotoxic T-lymphocyte-associated antigen 4 (CTLA-4). These processes, which normally maintain or restore immune homeostasis following immune clearance of a pathogen, are co-opted in tumors to restrict immune cell activation.

CPI monoclonal antibody immunotherapies are designed to interfere with checkpoint pathways that suppress T cell activation, thereby enhancing antitumor immune responses. Pioneering agents include ipilimumab (anti-CTLA-4), which was approved in 2011 for advanced melanoma,^4^ nivolumab, and pembrolizumab (anti-PD-1), which were approved in 2014,^4^^,^^5^ and atezolizumab (anti-PD-L1), which was approved in 2020.^6^ Clinical trials (e.g., CheckMate 067) have shown that ipilimumab and nivolumab combinations resulted in 5-y survival rates of 52% in advanced melanoma.^4^ However, monotherapies and, more commonly, their combinations often lead to a higher occurrence of immune-related adverse events (irAEs), including colitis, pneumonitis, endocrinopathies, and dermatologic toxicities, likely arising from heightened immune activation that targets nonmalignant tissues.^7^

The monoclonal antibody relatlimab (anti-LAG-3) was approved in 2022 in combination with nivolumab (marketed as Opdualag) for the first-line treatment of metastatic melanoma. This approval was based on the results of the RELATIVITIY-047 trial, which demonstrated improved progression-free survival for Opdualag compared to nivolumab monotherapy, establishing LAG-3 as a clinically validated checkpoint target in melanoma.^8^^,^^9^ These therapies have displayed significant clinical efficacy in advanced-stage disease, alongside the more recent approval of anti-PD-1 monotherapy also in the adjuvant setting.^5^^,^^6^

Despite these advances, approximately half of patients respond to CPI treatment, and many develop subsequent toxicity. Currently, it is not possible to predict which patients will benefit from CPI and which patients will experience irAEs. CPI immunotherapies have significantly improved patient prognoses compared with previous conventional treatments, yet, clinical challenges emphasize the need for further understanding of patient immune responses and for considering careful patient selection and clinical management. Given the diverse clinical responses to CPIs, and the lack of established predictive tools, ongoing research is dedicated to identifying biomarkers for predicting both treatment efficacy and potential toxicities.

Alongside T cells, B cells and their expressed antibodies form the adaptive immune system and may play key roles in immune regulation and treatment outcomes. B cells originate from hematopoietic stem cells in the bone marrow and mature within primary and secondary lymphoid organs. Upon activation, they differentiate into plasma cells that secrete antibodies or into memory B cells, which provide long-term memory and immune protection. While T cells have historically been the focus of melanoma immunology research, B cells are gaining increasing attention as important, albeit less well-studied, players in tumor immune responses.

In this review, we highlight emerging evidence on the regulatory and autoimmune phenotypes and functional roles of B cells in melanoma, and their associations with disease progression. We explore the growing evidence for the relevance of B cells in immunotherapy responses and B cell signatures that may be of predictive and prognostic significance, offering opportunities to monitor patients and improve clinical outcomes.

## Overview of B cell and antibody characteristics in melanoma

### B cells in cutaneous surveillance and antigen challenge

Skin-resident immune surveillance has been considered largely in the context of the T cell response; however, emerging evidence suggests that B cells can be recruited and locally proliferate in human skin.^10^ Upon antigen challenge of healthy skin, significantly increased B cell infiltration of memory B cells and plasmablasts has been reported, with some expressing the cutaneous lymphocyte antigen (CLA), normally associated with T cell skin homing propensity, indicating the recruitment of B cells with a skin-homing phenotype from the circulation.^10^ These suggest that in healthy skin, antigen challenge recruits and stimulates humoral immunity.

### B cell infiltrates in melanoma lesions

B cells are recruited to the tumor lesions of patients with cutaneous melanoma. Single-cell RNA sequencing and flow cytometry studies reveal heterogeneous B cell populations in melanoma lesions, including naïve and memory B cells, plasma cells, and regulatory B cells (Bregs).^11^^,^^12^ A study of 73 skin and cutaneous melanoma samples showed higher B cell frequencies in melanoma compared with normal skin, local B cell proliferation, antibody maturation, and likely unique antigen recognition repertoires in melanoma compared with nonmalignant skin. These suggest that melanoma can stimulate and reshape cutaneous humoral immunity.^13^

### B cell assembly in tertiary lymphoid structures (TLSs)

In tumor lesions, infiltrating B cells may assemble in clusters, sometimes developing into more organized tertiary lymphoid structures (TLSs); intratumoral lymphoid aggregates in which B cell diversification may occur. TLS are highly heterogeneous, ranging from cell clusters to follicular/germinal center-like formations. Higher frequencies of larger and more developed TLS with germinal center-like characteristics are often associated with advanced metastatic versus primary tumors.^14^^,^^15^ TLS-associated B cells express proinflammatory genes, including TNF, IL6, CXCL9, CXCL10, IFN, and IFNα/γ-related response signatures (IL-2/STAT5 pathway).^16^ These genes may participate in localized adaptive immune responses through antigen presentation, T cell activation and high-affinity, potentially self-antigen, and tumor antigen-reactive antibody production. B cells also synergize with follicular helper T cells (Tfhs) and dendritic cells (DCs),^16-21^ in TLSs, where B cells and DCs present antigens, promoting the formation of TCF7⁺ naïve/memory-like and cytotoxic CD8⁺ T cells to enhance and sustain antitumor immunity.^18^ Accordingly, several studies have demonstrated positive prognostic roles of B cells within TLS in melanoma, and correlations between TLS presence and immune activation with improved survival and CPI responses.^16^^,^^18^^,^^21^^,^^22^

### Distinct B cell differentiation signatures in patient blood

The circulation of patients with advanced melanoma versus healthy individuals features enrichment of naïve (CD21lo) B cells, double-negative (IgD^−^CD27^−^) B cells, and immunosuppressive (IL-10^+^CD95^+^) plasmablasts.^23^ CD21lo naïve B cells and double-negative (IgD^−^CD27^−^) B cells are characteristic of extrafollicular responses commonly described in autoimmune diseases such as systemic lupus erythematosus (SLE) and have been suggested as precursors to plasmablasts.^24^^,^^25^ Although plasmablasts are thought to exert rapid immune responses to pathogens, they can also acquire regulatory and exhausted phenotypes, characterized by secretion of IL-10 and expression of the death ligand receptor CD95. These features have been reported in B cells from patients with melanoma. A Th2 inflammatory environment may skew humoral responses to favor immune-inert and Th2 immunoglobulin isotypes, as demonstrated by higher serum levels of IgG4 and IgE in melanoma.^23^ IL-10 is linked to immunosuppression, reducing T cell activity, and potentially impairing antitumor responses and CPI therapy efficacy.^23^^,^^26^

### Distinct B cell characteristics in tumors versus patient blood

**Figure 1.**
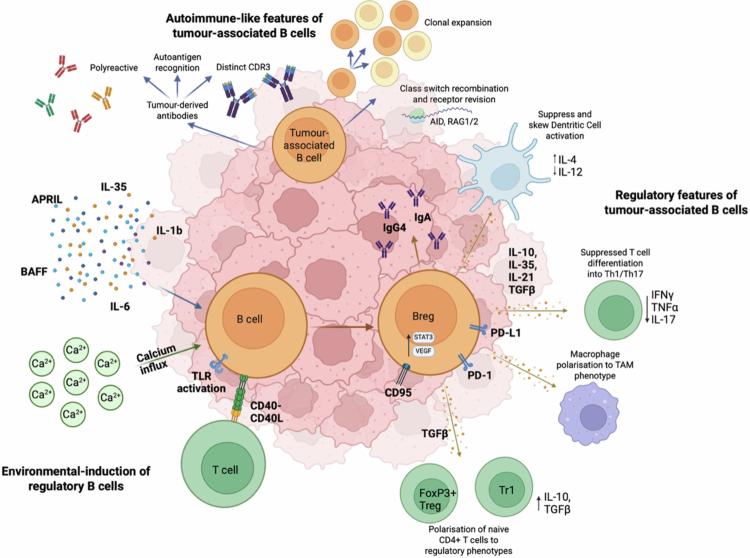
Melanoma-driven B cell dysregulation and autoimmune features. Several melanoma-induced and environmental factors may drive the expansion and differentiation of B cells into regulatory states. These factors may include calcium influx, TLR activation, and CD40L stimulation, which drive anti-inflammatory cytokine the production such as IL-10. Secreted cytokines such as IL-6, IL-1β BAFF, and APRIL may also influence Breg differentiation to promote IL-10 production, and regulatory cytokines such as IL-35 by B cells can skew Breg differentiation. Regulatory B cells are found at several stages through the differentiation spectrum. They possess immunosuppressive capacity via anti-inflammatory cytokine production (IL-10, IL-35, IL-21, and TGFβ), immune-suppressing surface and intracellular marker expression (PD-1, CD95, and VEGF), and may class switch to less effective antibody isotypes such as IgG4 and IgA. These immunosuppressive properties of Bregs may influence surrounding immune cells, such as the suppression of DC activation, the skewing of macrophage polarization to immunosuppressive phenotypes, the suppression of T helper differentiation, and the promotion of FoxP3+ Tregs in a TGFβ-dependent manner. Tumor-associated B cells may also possess autoimmune-like features. They have distinct CDR3 regions, undergo clonal expansion to produce tumor- or self-reactive B cells, and undergo class switch recombination and receptor revision, as shown by the presence of AID, RAG1, and RAG2 in the tumor.

Interestingly, a lower frequency of naïve B cells and higher abundance of differentiated and memory B cells in melanoma lesions has been reported compared to matched blood.^27^ Memory B cells enriched within the TME relative to matched peripheral blood exhibit distinct isotype distributions and antibody repertoires. Tumor-infiltrating B cells undergo clonal expansion, class-switch recombination, somatic hypermutation, and receptor revision, which is consistent with local, antigen-driven selection, likely through interactions with T cells within TLS and via extrafollicular pathways. Tumor-infiltrating B cells show elevated frequencies of unproductive immunoglobulin gene rearrangements compared to circulating B cells, reflecting impaired antibody production in the TME.^27^ Furthermore, tumor-associated B cells generate antibodies with distinct complementarity-determining region 3 (CDR3) architectures, increased polyreactivity, and autoantigen recognition.^27^ Collectively, these features indicate an active yet dysregulated, autoimmune-like humoral response in the tumor (Figure 1).

### Tumor-infiltrating plasma cells (PCs)

CD138-expressing PCs have been reported in melanoma; alongside B cells within tumors, these are commonly associated with better prognosis.^20^^,^^28^ However, one study of 710 melanomas studied PC infiltration in relation to histological prognostic markers: a PC-rich TME largely correlated with thicker, ulcerated tumors, and thus poorer prognosis.^15^ Tumor-infiltrating plasma cells expressed predominantly class-switched IgG and oligoclonal IgA antibodies, pointing to induction of an antigen-driven response.

### Association of B cell infiltrates and TLS with CPI responses

At baseline and on treatment, enrichment of B cells within tumors and TLS alongside upregulated proinflammatory gene signatures, is associated with improved immunotherapy responses.^16^^,^^19-21^^,^^29-34^ Higher frequencies of activated and memory B cell subsets are reported in CPI responders.^19^^,^^20^ TLS sites promote B cell interactions with T cells and myeloid cells, thus heightening antitumor immune responses and CPI efficacy.^12^^,^^19-21^ These support the premise that TLS-resident B cells may be critical for effective antitumor immunity, and correlate with improved clinical outcomes.

## Dysregulated B cell and antibody responses

### Regulatory B cells in melanoma

Melanoma may manipulate B cell function to promote regulatory states and induce immune tolerance. Several studies have outlined the phenotypic characteristics that define the immunosuppressive attributes of immunosuppressive and regulatory B cells in melanoma^35^^,^^36^ (Table 1). Typically, Bregs produce IL-10, TGF-β, IL-35, and other alternative Th2 immune mediators, and are shown to suppress T cell responses, DC activation, and inflammatory immune activity^37^ (Figure 1).

**Table 1.** Immunosuppressive and regulatory roles of B cells in patients with melanoma.

Context	Timepoint	CPI agent	Findings	Study
Peripheral blood and tumor biopsies	On-treatment and post-treatment	Anti-CTLA-4 (Ipilimumab) monotherapy and combination anti-CTLA-4 (Ipilimumab) + anti-PD-1 (nivolumab) therapy	Observed a decline in circulating B cells during treatment with combination CPI, alongside a proportional expansion of CD21lo B cells (expressing high levels of PD-1) and plasmablasts These changes were associated with several systemic irAEs, indicating a dual immunostimulatory and immunosuppressive role of B cells during CPI therapy	Das et al.^38^
Peripheral blood	Pre-treatment and on-treatment	Anti-PD-1 (nivolumab and pembrolizumab) monotherapy and anti-CTLA-4 (Ipilimumab) monotherapy	Showed that CPI induces broad humoral immune activation, including autoantibody formation against self-antigens Autoantibody-positive patients often developed irAEs reflecting dysregulated B-cell responses. Associations were found between the type of autoantibody developed on first CPI treatment (antithyroid) and subsequent irAE developed (thyroid dysfunction) during anti-PD-1 therapy Such overactivation may divert immune resources and promote systemic immune imbalance, reflecting a regulatory or immunosuppressive shift in humoral immunity during therapy	de Moel et al.^39^
Tumor and peripheral blood	Pre-treatment and on-treatment	Anti-PD-1 (nivolumab, pembrolizumab) monotherapy and combination anti-PD-1 (nivolumab) + anti-CTLA-4 (ipilimumab) therapy	Demonstrated that melanoma patients with high frequencies of Bregs expressing PD-L1, IL-10, and IgA showed reduced T-cell effector function and diminished responses to CPI. These Bregs were enriched in both tumor and circulation and correlated with nonresponse to therapy	Gatto et al.^40^
Tumor	Pre-treatment	Anti-PD-1 (nivolumab or pembrolizumab) monotherapy and combination anti-PD-1 (nivolumab) + anti-CTLA-4 (ipilimumab) therapy	Showed that tumor-infiltrating B cells (TIBs) in melanoma displayed regulatory phenotypes marked by high IL-10, PD-L1, and TIGIT expression These Bregs suppressed T-cell proliferation and IFNγ production and were significantly enriched in tumors from nonresponders to checkpoint blockade, indicating that regulatory B cells contribute to an immunosuppressive and therapy-resistant microenvironment	Ghosh et al.^41^
Peripheral blood and tumor	Pre-treatment	N/A	Observed enrichment of TGFβ+and PD-L1+ regulatory B cell populations and a reduction in TNFα+ B cell populations in melanoma patient circulation compared to healthy blood Ex vivo studies of patient-derived B cells promoted FoxP3+ Treg differentiation in a TGF-β-dependent manner, suggesting a B-T cell cross-talk with immunosuppressive attributes	Harris et al.^42^
Tumor	Pre-treatment	Anti-PD-1 (nivolumab or pembrolizumab) monotherapy and combination anti-PD-1 (nivolumab) + anti-CTLA-4 (Ipilimumab) therapy	Identified a subset of B cell and plasma cell gene signatures, including those linked to immune exhaustion and suppressive phenotypes, that were enriched in tumors of nonresponders to CPI These findings suggest that B cells with regulatory or dysfunctional features may contribute to immune resistance in melanoma	Johannet et al.^43^
Tumor and peripheral blood	Pre-treatment and on-treatment	Anti-CTLA-4 (Ipilimumab) monotherapy and combination anti-CTLA-4 (Ipilimumab) + anti-PD-1 (nivolumab) therapy	Identified subsets of IL-10+ B cells that form immune complexes and enhance myeloid cell-mediated immunosuppression. These Bregs dampened T-cell activation and contributed to an immunosuppressive tumor microenvironment in melanoma patients receiving CPI	Somasundaram et al.^11^
Peripheral blood	Pre-treatment and on-treatment	Anti-PD-1 (pembrolizumab/nivolumab) monotherapy	Found increased frequencies of IL-10+ plasmablasts, alternatively-activated DN B cells and elevated IgG4 and IgE in the circulation of melanoma patients compared to healthy subjects Increased baseline levels of these cells, alongside increased IgE and IgA were associated with a lack of toxicity, denoting reduced antitumor immunity in these patients Enrichment at baseline of PD-L1+/β Bregs, naïve B cells, and transitional B cells, as well as serum IgG4 and IgE was associated with reduced overall survival	Willsmore et al.^23^

Expansion and differentiation of Bregs can be triggered by several converging inflammatory cues such as TLR ligands, for example CpG/TLR9 or LPS/TLR4, and CD40 stimulation to induce expression of immunosuppressive cytokines such as IL-10.^37^^,^^44^ Tumor-associated B cells in metastatic melanoma have been reported to upregulate PD-L1 and stimulate the downregulation of major histocompatibility complex (MHC) molecules on melanoma cells, thereby contributing to immune escape^11^ (Figure 1).

In homeostasis, B cell receptor (BCR)-induced calcium influx via endoplasmic reticulum calcium sensors (STIM1/2) is essential for IL-10 production and Breg function.^45^ Inflammatory cytokines from barrier tissues and microbiota, especially IL-1β and IL-6, also promote IL-10-Bregs.^46^ TNF superfamily signals can push B cells into a regulatory state, for example, BAFF induces IL-10 producing Bregs via the transcription factor activator protein 1 (AP-1),^47^ and APRIL increases IL-10 and the suppressive function of human B cells^48^ (Figure 1).

Functionally, Bregs are a state rather than a fixed lineage, IL-10 producing B cells are reported to arise principally from transitional, naïve, memory, and plasmablast compartments depending on the immune context.^49-52^ In melanoma, single-cell and spatial analyses identified TGF-β+/PD-L1+ Bregs within tumors and blood, with a bias towards memory phenotypes, and evidence of engaging T cells and fostering Treg differentiation.^42^ This study investigated whether melanoma alters B cell functions to favor immune suppression by promoting TGF-β+/PD-L1+ Bregs and reducing proinflammatory B cells (TNFα+). This entailed evaluation of peripheral blood and tumor samples from melanoma patients alongside healthy subjects. The B cell compartment in melanoma patients exhibited a significantly higher frequency of circulating TGF-β+/PD-L1+ B cells, alongside a reduced number of TNFα+ B cells, compared with healthy individuals. These TGF-β+ B cells were identified across the B cell differentiation spectrum and were not associated with a specific lineage, therefore suggesting that their regulatory features were triggered in response to environmental stimuli. Furthermore, within the TME, TGF-β+ B cells were shown to form spatially organized clusters together with T cells and engage in crosstalk with T cells via chemokine signals. Patient B cells can induce FOXP3+ Tregs in a TGF**-**β dependent manner, while simultaneously supporting Tfh cell proliferation and cytokine secretion. reg induction was reversed by an anti-PD-1 antibody, pointing to a potential mechanism of targeting Bregs to enhance CPI treatments.^42^ In concordance, heightened Breg infiltration has been directly linked to reduced CPI efficacy and less favorable clinical outcomes,^53^ suggesting that targeting immunosuppressive B cell subsets may contribute to therapeutic responses.

Research to-date, therefore, highlights the diversity of B cells in melanoma and supports the notion that B cells likely contribute to a range of protumor and antitumor effects in the TME.^54^ However, tumor exposure often leads B cells to adapt to tolerogenic or protumor states, including the induction of Bregs and downregulated BCR signaling. This impairs their key roles in antigen presentation and antibody production.^55^ Whilst some B cell subsets contribute towards antitumor immunity, contrastingly, Breg subsets are involved in promoting immune suppression. The balance between proinflammatory B cells and immunosuppressive Bregs may be a critical determinant of disease progression and outcomes. Formulating strategies to inhibit Bregs, whilst importantly preserving proinflammatory B cell subsets may enhance the CPI response and significantly improve therapeutic outcomes.

### Dysregulated antibody isotype distribution

Emerging evidence has brought to light dynamic B cell class switching processes in melanoma, which favor alternatively-activated isotypes such as IgG4, which likely mediate immune suppression and are linked to poorer patient outcomes. IgG4 isotype antibodies possess limited Fc-mediated immune-stimulating capacity in comparison to IgG1. Furthermore, IgG4 antibodies undergo Fab arm exchange, whereby the exchange of two half-molecules derived from two distinct IgG4 antibodies forms an unstable bispecific antibody complex.^56^^,^^57^ These antibodies are unable to crosslink identical antigens, which further decreases the already limited capacity of IgG4 to trigger complement activation and immune effector functions.^58^ The limited immune-stimulating function of IgG4 was demonstrated with an IgG4 isotype antibody targeting the melanoma-antigen chondroitin sulfate proteoglycan 4 (CSPG4) introduced in human melanoma xenograft-challenged mice. IgG4 showed impaired ability to restrict tumor growth while the corresponding anti-CSPG4 IgG1 significantly restricted tumor growth in the presence of human immune effector cells in these mice.^59^

Several studies have focused on the distribution of immunoglobulin isotypes in melanoma patient serum, peripheral blood and in tumor lesions.^22^^,^^23^^,^^59^^,^^60^ In comparison to healthy individuals, significantly higher circulating IgG4 to total IgG ratios were found in patient blood, and higher IgG4 ratios were associated with worse progression-free survival and overall survival.^60^ Furthermore, an increased frequency of IgG4-expressing B cells was found in patients with early- and late-stage melanoma in comparison to healthy individuals. While IgG4+ B cells were sparse in healthy skin, 43% of primary melanomas had IgG4+ B cell infiltrates.^60^ In concordance, a proportional enrichment of serum IgG4 was reported in the serum of patients with advanced melanoma compared with healthy individuals.^23^ Furthermore, novel IgG4+ B cell subsets with regulatory and proangiogenic attributes have been reported in melanoma lesions and patient circulation.^22^

Some studies reported an enrichment of IgA expression in melanoma, with higher circulating IgA levels in patients with active disease compared to healthy individuals,^61^ reflecting a shift toward Th2-polarized immunity. Mechanistically, IgA can exert both protumor and antitumor roles. It can trigger effective Fc-mediated functions via the recruitment and activation of FcαRI (CD89) receptor-expressing neutrophils, inducing antibody-dependent cellular cytotoxicity (ADCC) and phagocytosis (ADCP). Furthermore, IgA can drive the maturation and proinflammatory programming of DCs that prime and activate cytotoxic CD8⁺ T cell responses. Additionally, IgA-mediated phagocytosis into DCs enables antigen cross-presentation to antigen-specific CD8+ T cells, driving their cytotoxic functions.^62-64^ However, sheets of IgA+ plasma cells in the TME are associated with a less favorable prognosis,^15^ and proportional enrichment of IgA is associated with poorer survival.^27^ These suggest context-dependent roles in melanoma in driving potent antitumor immunity but also correlating with features of poorer prognosis.

Taken together, these findings suggest that Th2-driven humoral responses manifested in elevated IgG4 serum levels and IgG4+ B cells in melanoma form part of immune evasion mechanisms that are associated with disease progression and worse patient outcomes. Dysregulated levels of IgG4, and perhaps IgA, even at early stages of the disease, indicate an opportunity to assess antibody isotype balance and dysregulation as prognostic indicators of tumor-associated immune modulation and potential targets for restoring antitumor immunity.

### Autoimmune-like features of the humoral response in melanoma

Advanced stage III/IV melanoma has been shown to harbor altered B cell subsets and antibody repertoires.^23^ To investigate autoimmune responses, serum IgG antibodies from patients versus healthy volunteers were screened against a pool of human tissue extracts via immuno-mass spectrometry.^23^^,^^27^ This identified several autoantigens uniquely recognized by circulating antibodies in melanoma patients exclusively,^27^ some of which are reported in autoimmune diseases, IgG4-related disease, and cancer. Antibodies to eleven autoantigens significantly increased (including tubulin family members), and the levels of antibodies to fourteen antigens were decreased compared to controls, suggesting an altered autoimmune profile in patients.^23^ Autoantibody levels against tubulins and other antigens were significantly higher in patients with active disease compared to those with resected disease or healthy volunteers, which is consistent with elevated tubulin gene expression in melanoma tissue. These highlight the possibility of identifying humoral response biomarkers associated with disease progression.

CyTOF analysis of matched peripheral blood mononuclear cells demonstrated correlations between autoantibody reactivity to β1-tubulin (TUBB1) and class-switched IgG+ memory B cells, as well as exhausted suppressive CD4+ and CD8+ T cell clusters, linking humoral autoreactivity to dysfunctional cellular immunity.^27^ Antibodies against the histone protein H4C1 correlated with IL-10+ plasmablasts, further pointing to the autoimmune-like nature of these responses.^27^ Within tumors, B cells exhibited clonal expansion, class switching, somatic hypermutation, and receptor revision, producing antibodies with higher rates of unproductive sequences and unique hypervariable region characteristics compared to circulating B cells.^27^ These indicate affinity maturation and polyreactivity, which is consistent with an aberrant autoimmune-like response within the TME.

These findings reveal that melanoma may foster autoimmune-like B cell and antibody responses characterized by polyreactivity and autoantigen recognition. Such dysregulated humoral immunity mirrors extrafollicular B cell activity in autoimmune diseases,^24^ perhaps reflecting enhanced regulatory responses, aberrant differentiation or immune exhaustion, mechanisms also reported in non-small cell lung cancer and squamous cell carcinoma.^65^^,^^66^ Understanding the polyreactive and autoantigen-recognizing nature of tumor-derived antibodies could inform strategies to modulate B cell activity or exploit their unique repertoires for biomarker development and therapeutic antibody engineering in cancer immunotherapy.

## Features of humoral immunity as biomarkers of CPI outcomes

### Baseline B cell signatures and autoimmune features may predict clinical responses to CPI

Emerging evidence indicates a pivotal role for B cells and humoral immunity in responses to CPI immunotherapy (Table 2).

**Table 2.** Predictive roles of B cell signatures in response to checkpoint inhibitor immunotherapy.

Context	Timepoint	CPI agent	Findings	Study
Tumor	Pre-treatment	Anti-PD-1 (pembrolizumab or nivolumab) monotherapy and Anti-CTLA-4 (Ipilimumab) monotherapy	Presence of tertiary lymphoid structures (TLS) and B cell–rich immune niches in melanoma associated with improved patient survival and better responses to CPI	Cabrita et al.^18^
Tumor	Pre-treatment	Anti-PD-1 (nivolumab or pembrolizumab) monotherapy, and combination anti-PD-1 (nivolumab) + anti-CTLA-4 (Ipilimumab) therapy	The presence of B cell-related immune gene signatures was associated with improved immunotherapy response, reporting pathway enrichment of proinflammatory TNFα and IFNγ in these responding patients	Egan et al.^31^
Tumor	Pre-treatment	Anti-PD-1 (nivolumab, pembrolizumab) and anti-CTLA-4 (Ipilimumab), including combination nivolumab + Ipilimumab therapy	Increased B cells and presence of tertiary lymphoid structures (TLS) in tumors at baseline correlated with significantly improved clinical response and overall survival to CPI therapy	Helmink et al.^16^
Tumor	Pre-treatment	Anti-PD-1 (nivolumab or pembrolizumab) monotherapy and combination anti-PD-1 (nivolumab) + anti-CTLA-4 (Ipilimumab) therapy	Identified immune subtypes in cutaneous melanoma with high B cell infiltration and TLS signatures that were strongly correlated with enhanced immune activation and improved response to CPI; B cell-rich subtype showed best prognosis	Liu et al.^34^
Tumor	Pre-treatment	Anti-PD-1 (nivolumab or pembrolizumab) monotherapy, anti-PD-L1 (atezolizumab) monotherapy, and combination anti-PD-1 (nivolumab) + anti-CTLA-4 (Ipilimumab) therapy	Developed a B cell-related gene signature that predicted improved response to CPI across multiple cancer types (melanoma, RCC, and NSCLC) High B cell signature scores were associated with better survival and higher objective response rates in patients with melanoma.	Lundberg et al.^33^
Tumor	Pre-treatment	Anti-PD-1 (nivolumab or pembrolizumab) monotherapy and anti-CTLA-4 (Ipilimumab) monotherapy	Heterogeneity in TLS-associated B cell phenotypes (including naïve, memory, and plasma-like subsets) correlated with patient survival in metastatic melanoma treated with CPI Higher proportions of memory-like B cells in TLS were linked to improved outcomes.	Lynch et al.^20^
Tumor	Pre-treatment	Anti-PD-1 (nivolumab) monotherapy	High levels of IGK-expressing plasma cells at baseline were associated with responders to anti-PD1 therapy	Onieva et al.^30^
Tumor	Pre-treatment	Anti-PD-1 (nivolumab or pembrolizumab) monotherapy and anti-CTLA-4 (Ipilimumab) monotherapy	Higher B cell gene expression (including CD19, CD20, and IG-related transcripts) in pre-treatment melanoma tumors is associated with improved overall survival Memory B cell signatures were associated with improved survival, and regulatory B cells were linked to worse prognosis Lack of BCR assembly in baseline tumors was linked to poor response to anti-CTLA-4	Selitsky et al.^53^
Peripheral blood	Pre-treatment	Anti-PD-1 (nivolumab or pembrolizumab) monotherapy	Baseline B cell signatures were used to predict toxicity in patients receiving anti-PD1 therapy Enrichment of double-negative B cells and plasmablasts, and high IgE and IgA was associated with a lack of toxicity, suggesting a protective role	Willsmore et al.^23^
Tumor	Pre-treatment	Anti-PD-1 (nivolumab or pembrolizumab) monotherapy and anti-PD-L1 (atezolizumab) monotherapy	CD20⁺CD22⁺ADAM28⁺ B cells within tertiary lymphoid structures promoted enhanced responses to CPI These B cells facilitated antigen presentation and T cell activation, contributing to improved therapeutic outcomes across multiple cancer types	Wu et al.^19^

**Figure 2.**
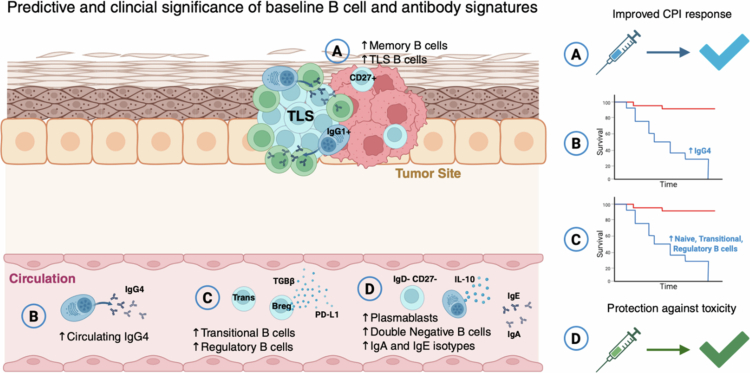
B cell and antibody signatures of treatment-naïve patients may harbor predictive and clinical significance for patient prognosis and immunotherapy treatment response. Association between B cell signatures in patients with melanoma prior to treatment and clinical responses. (A) Enrichment of memory B cells and TLS-associated B cells in melanoma lesions is associated with an improved response to CPI. (B and C) whereas upregulated circulating naïve, transitional, and regulatory B cells and the IgG4 subclass immunoglobulins are correlated with worse survival outcomes. (D) Increased circulating plasmablasts, double-negative B cells, and alternatively activated Ig isotypes IgA and IgE are protective against toxicity during CPI.

Several studies have shown that, prior to treatment, enrichment in B cells, particularly of class-switched memory subsets, within tumor lesions were associated with improved responses to CPI^12^^,^^16^^,^^29^ (Figure 2). This association denotes pre-existing activated humoral immunity within the tumor that may be primed for a more effective response once checkpoint blockade is initiated. Furthermore, the spatial organization, maturation state, and density of TLS-associated B cells within tumors have been linked with more favorable progression-free and overall survival and an enhanced response to anti-PD-1 immunotherapy^16^^,^^18^^,^^20^^,^^21^ (Figure 2).

B cell profiles may offer opportunities to predict treatment outcomes, aid in patient selection, and guide therapeutic decisions. A study of circulating B cell and antibody signatures at baseline prior to anti-PD1 immunotherapy showed that enrichment of class-switched (IgG+) memory B cells was associated with better overall survival following treatment, whilst higher frequencies of naïve, transitional and regulatory (TGF-β+/PD-L1+) B cells and antibody isotypes IgG4 were associated with worse overall survival^23^ (Figure 2). Furthermore, assessment of baseline B cell signatures, pointed to enrichment of IL-10-expressing plasmablasts and double-negative (CD27^−^IgD^−^) B cells, as well as nonclassical antibody isotypes IgA and IgE in the circulation, to be protective against the subsequent development of irAE during anti-PD-1 therapy^23^ (Figure 2).

**Figure 3.**
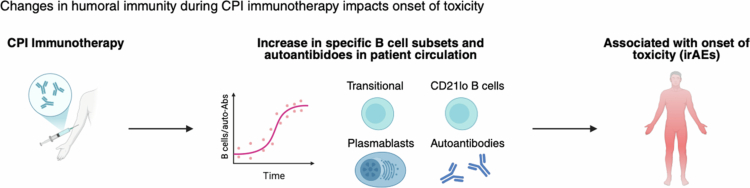
Dynamic changes in the phenotypes of circulating B cells during immunotherapy and association with the development of toxicity. Alterations in humoral responses on treatment include the proportional increase in transitional and CD21lo B cells, plasmablasts, and autoantibodies, which are associated with higher probability of the onset of toxicity (immune-related adverse events, irAEs) during CPI.

Furthermore, baseline serum autoantibody signatures have been linked to both therapeutic efficacy and irAE development in specific tissues^23^^,^^39^^,^^41^^,^^43^ (Figure 3). One study using a microarray to profile serum autoantibodies reported that patients with high pretreatment autoantibody levels were associated with reduced recurrence-free survival; a multivariable analysis revealed that high baseline autoantibody levels predicted severe toxicity.^43^ However, Ghosh et al.^41^ reported that high baseline autoantibodies correlated with reduced irAE development on treatment with CPI, suggesting potential protective roles.^41^ It is possible that autoreactive B cells and antibodies may both modulate toxicity (irAE protection) and shape therapeutic efficacy under CPI.

Together, baseline enrichment of class-switched memory B cells correlates with improved outcomes in immunotherapy, while higher frequencies of naïve, transitional, and PD-L1⁺ TGFβ⁺ regulatory B cells predict poorer outcomes. Additionally, serum autoantibody profiles and IL-10⁺ plasmablast signatures may modulate both therapeutic efficacy and immune-related toxicity, highlighting humoral immunity as a dual biomarker for CPI benefit and safety.

### Impact of CPI treatment on the humoral compartment in patient blood and tumors

**Figure 4.**
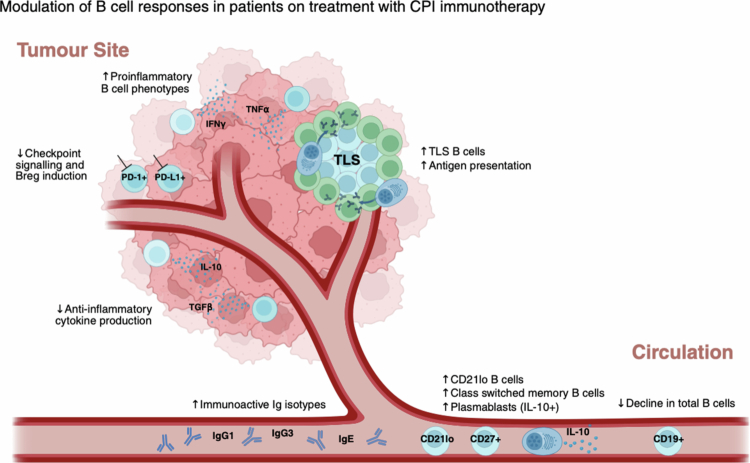
Checkpoint inhibitor immunotherapy impacts B cell and antibody signatures in the tumor and circulation of patients with melanoma. On treatment with CPI, circulating B cells have been reported to decline, whilst specific B cell subsets proportionally increase; these include naïve CD21lo B cells, class-switched memory B cells and plasmablasts. The circulating immunoglobulin isotype repertoire also demonstrates a dynamic shift on treatment, favoring mature, and immunoactive isotypes such as IgG1, IgG3, and IgE. In tumors, checkpoint inhibitors stimulate proinflammatory B cell signatures such as IFNγ and TNFα, as well as increased TLS-associated B cells and antigen presentation. Checkpoint signaling, Breg induction, and anti-inflammatory cytokine production in the tumor are reduced during CPI treatment.

CPI treatment is designed to trigger antitumor immune responses via reactivation of exhausted T cells. However, emerging evidence points to a significant impact on the humoral compartment. Longitudinal studies have shown that treatment with CPI impacts the frequency, activation status, and phenotypic profiles of circulating B cells and antibodies (Figure 4).

One study used peripheral blood samples from 39 patients treated with either anti-PD-1 or anti-CTLA-4 monotherapy or combination. Despite a reduction in circulating B cells after one cycle of combination CPI, a proportional enrichment of CD21lo B cells, memory B cells, and plasmablasts was found within the remaining circulating B cell pool.^38^ Consistent with these results, another study reported increased proportions of circulating class-switched memory B cells and IL-10-expressing plasmablasts during anti-PD-1 treatment in 52 patients with advanced (grades III and IV) melanoma. This was accompanied by a distinct shift in the serum immunoglobulin isotype repertoire to favor mature, immunoactive isotypes such as IgG1, IgG3, and IgE on treatment.^23^ These were reported alongside reduced antibody isotypes associated with lower effector functions, IgG2, IgG4, IgA, and IgM, and decreased tubulin-targeting autoantibodies. These studies reveal a dynamic change in B cell signatures supporting the expansion of class-switched memory B cells, rapidly responding plasmablasts, and immunoactive antibody isotypes during CPI treatment, which may promote effective antitumor responses. However, CPI treatment has also been shown to trigger the proliferation of atypical, exhausted or regulatory B cell subsets, such as IL-10+ CD95hi plasmablasts and IgG-IL-10+ CD95hi memory B cells,^23^ thus highlighting the potential for B cell exhaustion and B cells with limited antitumor potency.

Moreover, differences in B cell abundance and phenotypes within the TME are observed during CPI immunotherapy (Figure 4). On-treatment tumor biopsies demonstrate an increased presence of functionally-activated and clonally-expanded B cells within the tumors of responders and enrichment of B cell subsets and BCR repertoire changes that correlate with clinical benefit.^16^ These might reflect reduced immunosuppression following CPI, allowing B cells, often within TLSs, to facilitate antigen presentation and stimulate antitumor responses.^18^ Within TLSs, Tfh cells promote germinal center B cell selection and high-affinity antibody generation, and through IL-21 can support CD8+ T cell proliferation, viability, cytokine secretion, and cytotoxicity. Tfh abundance is also linked to anti-PD-1 efficacy in experimental tumor models and improved outcomes in patients.^16^^,^^18^^,^^67^^,^^68^

Furthermore, CPI therapy may mitigate the immunosuppressive effects of Bregs, shifting the balance toward more functionally activated and proinflammatory B cell phenotypes. This may be achieved through several mechanisms including: direct blockade of PD-1/PD-L1 signaling on PD-L1 or PD-1 expressing Bregs which may favor a more activated proinflammatory state^69^ and a reduction of immunosuppressive (e.g., IL-10 and TGF-β) cytokine production known to suppress CD8+ T cells, induce Tregs and inhibit other antigen-presenting cells.^70-72^ As a result, CPI therapy may reduce checkpoint signaling and lower Breg induction and production of IL-10 and TGF-β.^27^ Furthermore, Bregs rely on co-inhibitory molecules such as TIM-1 and TIGIT, to maintain immunosuppressive features, such as IL-10 production.^73^ Whilst these are not yet targetable checkpoints, preclinical studies suggest that blocking TIM-1 or TIGIT may harbor the potential to reverse Breg-mediated immunosuppression.^74^^,^^75^ In the future, combination treatments may be designed to stimulate the B cell arm of adaptive immunity. Therefore, during CPI, B cell compartment evolution towards memory B cell phenotypes may promote improved antigen presentation and antibody production, leading to better clinical responses.

Conversely, there is evidence for dynamic B cell phenotypic and functional shifts on treatment being associated with the onset of toxicity. On-treatment enhanced circulating levels of overall (CD19+) and transitional (CD19+CD10+CD24^high^CD38^high^) B cells are associated with the development of inflammatory arthritis irAEs.^40^ Similarly, a decline in circulating B cells and a proportional increase in CD21lo B cells and plasmablasts were associated with the development of irAEs and worse overall survival in patients who developed high-grade toxicity^38^ (Figure 3).

Longitudinal analyses further showed that CPI actively remodels the humoral compartment through autoantibody production on treatment. Increased serum autoantibodies to type II collagen during treatment were associated with CPI-induced inflammatory arthritis (IA).^40^ Similarly, on-treatment seroconversion of antithyroid autoantibodies has been linked to organ-specific toxicities during CPI.^39^

Collectively, these studies reposition B cells and their antibodies from passive bystanders to actively evolving components and potentially determinants of CPI efficacy and toxicity.

## Discussion: translational opportunities for immunotherapy

Our evolving understanding of B cells and antibodies in melanoma highlights multiple functions and may provide a valuable source of potential biomarkers, drawing on several distinct features: (1) lower naïve and higher memory/differentiated B cells in tumors vs blood^27^; (2) Class-switching and repertoire skewing, including enrichment of less immunoactive isotypes such as IgG4 that impair immune effector stimulation and antagonize IgG1 functions^13^^,^^59^^,^^60^; (3) Higher nonproductive immunoglobulin rearrangements and distinct CDR3 in tumor-associated B cells^27^; (4) Melanoma-reactive and autoantigen-reactive tumor and circulating antibodies^23^^,^^27^; (5) Intratumoral TLSs may indicate a primed in situ immunity and correlate with survival and immunotherapy benefit^18^; and (6) Regulatory IL-10⁺ B cell subsets may predict outcomes and toxicities under CPI. Several but not all features are shared with other immunogenic tumors; e.g., collapsed circulating memory B cells and increased isotype-switched CD20⁺CD27⁺IgD^−^ B cells; and the presence of TLS in triple-negative breast cancers (TNBC)^54^^,^^76^^,^^77^; and circulating IL-10⁺ Breg deficiency limits self-reactive T cell activity and autoantibody formation in advanced non-small cell lung cancer, predisposing to high-grade irAEs.^78^

Melanoma skews B cell immunity toward regulatory and exhausted states, including TGF-β⁺/PD-L1⁺ Bregs across the differentiation spectrum, that suppress antigen presentation, foster FOXP3⁺ Treg induction, and are spatially organized with T cells in tumors; these effects can be partially reversed by anti-PD-1.^42^ Concurrently, humoral responses show isotype dysregulation such as enrichment of IgG4, which possesses weak Fc effector function and is linked to worse progression-free and overall survival, while IgG1 retains superior antitumor activity.^23^ Autoimmune features are evident in elevated IL-10⁺ plasmablasts, double-negative B cells, and serum autoantibodies targeting multiple antigens, including β1-tubulin, with reactivity correlating to class-switched IgG⁺ memory B cells and exhausted CD4⁺/CD8⁺ T cell clusters.^23^ Within tumors, B cells undergo clonal expansion, class-switching, somatic hypermutation, and receptor revision, yielding antibodies with distinct CDR3 properties, higher unproductive sequences, and polyreactivity, consistent with an aberrant autoimmune-like response.^27^ The balance between proinflammatory B cells and immunosuppressive Bregs, coupled with isotype (IgG4/IgA) skewing and autoantibody signatures, may influence melanoma outcomes; targeting Bregs and correcting isotype bias while preserving antitumor B cell functions may represent a rational strategy.

Despite the recognition of regulatory roles for B cells, global B cell depletion has not shown conclusive clinical benefit. One study evaluated the anti-CD20 monoclonal antibody rituximab, intended to target CD20⁺ melanoma subpopulations with stem cell-like, tumor-initiating properties that might drive recurrence; this would also widely deplete B cells. Rituximab was well-tolerated, and several patients remained recurrence-free long after treatment, suggesting a potential benefit.^79^ In a pilot trial, anti-CD20-mediated B cell depletion showed signs of antitumor activity in 8 of 10 patients with treatment-resistant metastatic melanoma.^11^ However, rituximab as an adjunct to IL-2 therapy resulted in no improvement in response or toxicity compared to IL-2 monotherapy.^80^ These data were derived from small, nonrandomized cohorts. Thus, evidence supporting overt B cell targeting in melanoma remains limited, which is consistent with the mixed biology of humoral immunity, which can be proinflammatory/antitumor (e.g., TLS-associated benefit) or protumor.

Increased baseline levels of circulating tumor-reactive antibodies and memory B cell subsets are linked to positive outcomes and CPI responses. These effects may promote antitumor effects via mechanisms such as ADCC, ADCP, and complement activation. Their presence in melanoma not only signifies an active immune response but also implies a pre-existing ability for effective tumor recognition that can be harnessed in an appropriate therapeutic setting. Higher baseline densities of circulating memory B cells, tumor-associated B cells, and TLS-assembly features are significantly associated with improved responses to CPI therapy.^16^^,^^29-31^^,^^33^^,^^81^ This highlights opportunities to identify patients a priori who respond differentially to treatment. In contrast, enrichment of less activated and regulatory B cell subsets may indicate a bias toward alternatively activated and immunosuppressive humoral compartment phenotypes in melanoma and thus may denote worse patient prognosis.^53^ Specific B cell subsets and antibody isotypes appear protective against the development of toxicity, denoting various functional checkpoint roles for B cells and antibodies linked with the wide spectrum of CPI clinical outcomes.^23^ Identification of these predictive humoral signatures prior to treatment may facilitate prompt early intervention with combination strategies or a change in therapeutic agent to moderate, prevent, or avoid toxicity and improve outcome.^12^ Thus, autoimmune-linked B cell responses represent a dual-edged biomarker: predictive of survival and treatment outcomes, while highlighting potential targets for refining immunotherapy in melanoma.

Beyond baseline predictions, monitoring B cells and antibodies could reveal early or temporal indicators of clinical outcomes, immunotherapy effectiveness, and associated toxicity,^23^^,^^38^^,^^40^^,^^82^ offering dynamic biomarkers for monitoring. Serial assessments of circulating antibody titers and B cell subset abundance can provide real-time feedback on the efficacy of CPI. Increases in tumor-reactive antibody levels, for example, have been correlated with improved clinical outcomes and can serve as an early indicator of response.^16^^,^^18^ Dynamic monitoring of biomarkers is highly desirable and urgently needed for the prediction and early identification of CPI-associated toxicities. Both baseline phenotypes and changes in B cell compartment composition on-treatment may denote the emergence of irAEs. This real-time monitoring may allow swift adjustment of therapeutic regimens, thereby reducing the severity of irAEs.^7^

Dissecting humoral response evolution, the interactions of B cells and their antibodies with other immune and cancer cells, are pivotal to understanding how we may harness wider adaptive immunity to fight cancer. Unravelling B cell response mechanisms is vital for refining therapeutic strategies that could potentially further enhance B cell-mediated antitumor immunity whilst avoiding the induction of exhausted features and irAEs. These include pairing CPIs with agents that selectively deplete suppressive B cells, modulate cytokine signaling, or enhance B cell activation and differentiation to optimally harness B cell-mediated antitumor functions.

In clinical practice, implementing longitudinal monitoring of the humoral response necessitates standardized assays and validated thresholds. Recent progress in high-throughput RNA sequencing and multiparameter flow and mass cytometry technologies has enabled detailed characterization of B cell repertoires and antibody profiles. These advancements create ample opportunities to interrogate, establish, and implement biomarkers for melanoma disease progression or immunotherapy response, and optimize treatment schedules. Ultimately, understanding, considering, and harnessing the humoral immune response in the development of immunotherapy may offer critical insights into the significant proportion of patients who currently do not benefit from CPI treatments.

## Data Availability

Data sharing is not applicable to this article, as no new data were created or analyzed in this study.
